# P19 How do risk assessment models impact 30 day unplanned hospital readmissions in adult patients receiving outpatient parenteral antimicrobial therapy (OPAT)? A systematic review

**DOI:** 10.1093/jacamr/dlaf239.023

**Published:** 2026-01-05

**Authors:** T M Finnegan, A Phelan, B J O’Kelly

**Affiliations:** Community Intervention Team, Louth, Ireland; Trinity College, Dublin, Ireland; Our Lady of Lourdes Hospital, Drogheda, Ireland; Royal College of Surgeons in Ireland, Ireland

## Abstract

**Background:**

Outpatient parenteral antimicrobial therapy (OPAT) enhances health-related quality of life; however, unplanned readmissions may reduce patient satisfaction and the cost-effectiveness of the treatment. By predicting and minimizing these unplanned readmissions, patient outcomes can be significantly improved. Predictive models for unplanned OPAT readmissions can help identify high-risk patients, enabling tailored care and support. However, data on the effectiveness of OPAT-specific models is limited. This systematic review assessed the impact of risk assessment models on 30 day unplanned hospital readmissions among adult OPAT patients.

**Methods:**

A systematic review aiming to measure the impact of readmission risk models on 30 day unplanned hospital readmissions in adult OPAT patients. Searching CINAHL, Medline, Embase, ASSIA and Web of Science identified 435 potential articles. Independent selection, data extraction, tabulation of findings, and analysis were completed. Meta-analysis was conducted using JBI Sumari. When a meta-analysis was not feasible due to inappropriate data formats, the results were reported narratively.

**Results:**

Three studies met the inclusion criteria. The primary outcome of the review was to measure the impact of OPAT readmission risk models on 30 day hospital readmissions by evaluating their ability to accurately predict readmissions within 30 days. Two studies reported low c-statistics, indicating poor discriminative capability, while one study showed a c-statistic of 0.75 in a temporal validation cohort, suggesting modest discriminative ability but lower accuracy in a broader group. A meta-analysis of the likelihood of unplanned readmissions in OPAT patients found no significant differences in unplanned readmissions. Across the studies, the most common reason for readmission was infection-related, followed by adverse drug events and complications from vascular access devices. There were few reported readmissions for diarrhoea. The studies identified different risk factors for readmission depending on the patient case mix, the structure of the OPAT service, and the methods of OPAT delivery. Aminoglycosides and daptomycin were linked to a lower likelihood of readmissions, while other factors showed no clear trends. Results on endovascular infections and concurrent antimicrobial use had low heterogeneity indicating that the results can be trusted, but findings regarding drug-resistant organisms, age, Charlson Comorbidity Index, and previous admissions should be interpreted with caution due to high heterogeneity.

**Conclusions:**

The most common reason for readmissions was infection related. The review did not definitively identify risk factors for readmission and the impact of the OPAT readmission risk models was inconclusive. Further research is needed to enhance the transportability of these models and to develop versions tailored to different settings.

